# Precision in kidney‐sparing surgery: Robot‐assisted ureterectomy with novel Black Eye™ Ink

**DOI:** 10.1002/bco2.502

**Published:** 2025-02-19

**Authors:** Hayder Alhusseinawi, Naomi Nadler, Helene Reif Andersen, Juan Luis Vásquez, Thomas Norus, Nessn Azawi

**Affiliations:** ^1^ Department of Clinical Medicine Aalborg University Hospital Aalborg Denmark; ^2^ Department of Urology Gødstrup Hospital Gødstrup Denmark; ^3^ Department of Urology Zealand University Hospital Roskilde Denmark; ^4^ Institute for Clinical Medicine University of Copenhagen Copenhagen Denmark

**Keywords:** kidney sparing surgery, KSS, radical nephroureterectomy, RNU, segmental ureterectomy, UTUC

## Abstract

**Objective:**

To investigate the feasibility, oncological efficacy and safety of robotic segmental ureterectomy (SU) for treating patients with localised upper tract urothelial carcinoma (UTUC). A key aspect of this research involves utilising Black Eye™ Endoscopic Marker Ink to delineate the boundary of the tumour in the ureter, helping to ensure precise surgical intervention and reducing the risk of positive surgical margin.

**Patients and Methods:**

In a prospective non‐randomised trial from January 2018 to December 2022, patients with localised UTUC confirmed by CT‐urography were enrolled. A Multidisciplinary Team assessed patients for suitability for kidney‐sparing surgery (KSS) with SU, marked by endoscopic Black Eye™ Endoscopic Marker Ink. Black Eye Endoscopic Marker Ink marking aimed to enhance surgical precision by delineating clear resection margins. The primary endpoints were the feasibility of the technique, local and bladder recurrence rates and surgical outcomes. Propensity score matching was used for a balanced comparison to the standard treatment Radical Nephroureterectomy (RNU).

**Results:**

Thirty patients underwent SU, in the period of study with only one local recurrence reported with a median follow‐up time of 35 months. SU was associated with a significantly shorter operative time (41 minutes less on average, *p* < 0.001) than RNU. Tumour size was significantly larger in the RNU group (median size 42.5 mm, IQR: 30–60.5) compared to the SU group (median size 30 mm, IQR: 20–35) (*p* = 0.007), potentially indicating selection bias towards RNU for more advanced cases. No significant difference between the groups was found in the post‐operative Clavien‐Dindo complication score nor in oncological outcomes.

**Conclusion:**

SU with Black Eye™ Endoscopic Marker Ink marking is a viable KSS technique that offers a safe and effective alternative to RNU for patients with a single tumour, no longer than 30 mm and of low grade. This novel approach is promising in lowering the risk of positive margins, ensuring cancer control and preserving renal function.

## 1 INTRODUCTION

The landscape of urothelial carcinomas (UCs) is predominantly occupied by bladder cancer (BC), leaving upper urinary tract urothelial carcinomas (UTUCs) as a relatively rare entity, accounting for a mere 5–10% of all UC cases.^1^ In the Western hemisphere, the incidence rate hovers around two individuals per 100 000 annually. Within this spectrum, pyelocaliceal tumours appear with double the frequency of their ureteral counterparts, and approximately 10–20% of cases exhibit multifocal growths.^2^ Concomitant BC is present in 17% of UTUC cases, while a history of BC is observed in 41% of cases.^3^ A predominant male demographic, accounting for over 70% of UTUC cases, has been identified, with the majority being diagnosed following symptomatic manifestations, particularly visible haematuria.^4^

UTUCs tend to present with a higher rate of invasive disease at the time of diagnosis. Approximately two‐thirds of cases are compared to bladder tumours, where invasive presentations account for 15–25%. This disparity may stem from the lack of a muscularis propria layer in the upper urinary tract, predisposing these tumours to early upstaging.^5^

Radical nephroureterectomy (RNU) is regarded as the gold standard treatment for UTUC. Nonetheless, the procedure is not without serious complications, notably the impairment of renal function, which can significantly affect oncological prognoses. This concern has galvanised a shift toward nephron‐sparing modalities, aiming to strike a balance between cancer control and preserving renal integrity. Consequently, various kidney‐sparing surgeries (KSS) for managing low‐grade and limited‐volume UTUCs, including segmental ureterectomy, endoscopic resection and intraluminal therapy, have been explored and documented in clinical settings. KSS has emerged as a favourable alternative that diminishes the morbidity typically linked with more radical procedures, such as the loss of renal function while maintaining oncological effectiveness.^6^

Segmental ureterectomy (SU) with wide margins is particularly noteworthy, offering a strategic advantage for staging and grading the disease while conserving the ipsilateral kidney.^7^ For low‐risk tumours located in the distal ureter that is not amenable to complete endoscopic removal or for high‐risk tumours where KSS is imperative, complete distal ureterectomy with neo‐cystostomy is recommended.^7^, ^8^

One major challenge with SU is the difficulty in accurately identifying the ureter tumour, potentially leading to incomplete tumour resection and higher failure rates. Our study aims to introduce a novel methodology, for the first time, employing a Black Eye™ Endoscopic Marker Ink marker on the ureter to enhance the success rate of SU and to mitigate recurrence during robot‐assisted KSS. This innovative approach is anticipated to improve surgical precision and potentially optimise the equilibrium between oncological control and renal preservation.

## 2 MATERIALS AND METHODS

We began with animal experiments to establish the appropriate dose of Black Eye™ Endoscopic Marker Ink for injection into the ureter, aiming to minimise the risk of excessive ink spillage into the surrounding tissue.

Patients referred to our department of urology with suspected UTUC between January 2018 and December 2022 were invited to participate in the project.

All patients underwent standard procedure with CT‐urography, flexible cystoscopy and diagnostic ureteroscopy biopsies. To qualify for SU, individuals needed to have a single tumour that was no larger than 4 cm with low grade, located anywhere in the ureter and not appropriate for treatment with local laser therapy. All patients were evaluated by a Multidisciplinary Team (MDT) to determine their suitability for SU. Patients who were technically suitable for the study underwent an injection with Black Eye™ Endoscopic Marker Ink right before the SU. Patients unsuitable for segmental resection, or who declined this option, were asked to consent for their clinical data to be used in the control arm, receiving the standard RNU.

The study was approved by the regional ethical committee with register number SJ‐754.

The aim of the study was to assess the feasibility, oncological efficacy and safety of SU compared to RNU, with a specific focus on oncological outcome.

### 2.1 Animal experiment

In preparation for the clinical phase of our research, we carried out an initial experiment using animals to fine‐tune the dosage and method of applying an endoscopic ink marker. We chose a pig for this study, specifically focusing on its ureters to refine our technique. We used injeTAK® Cystoscopy Needles, 4.8 French in diameter and 45 cm in length to carefully injected the ink directly into the ureters through a ureteroscope.

**FIGURE 1:**
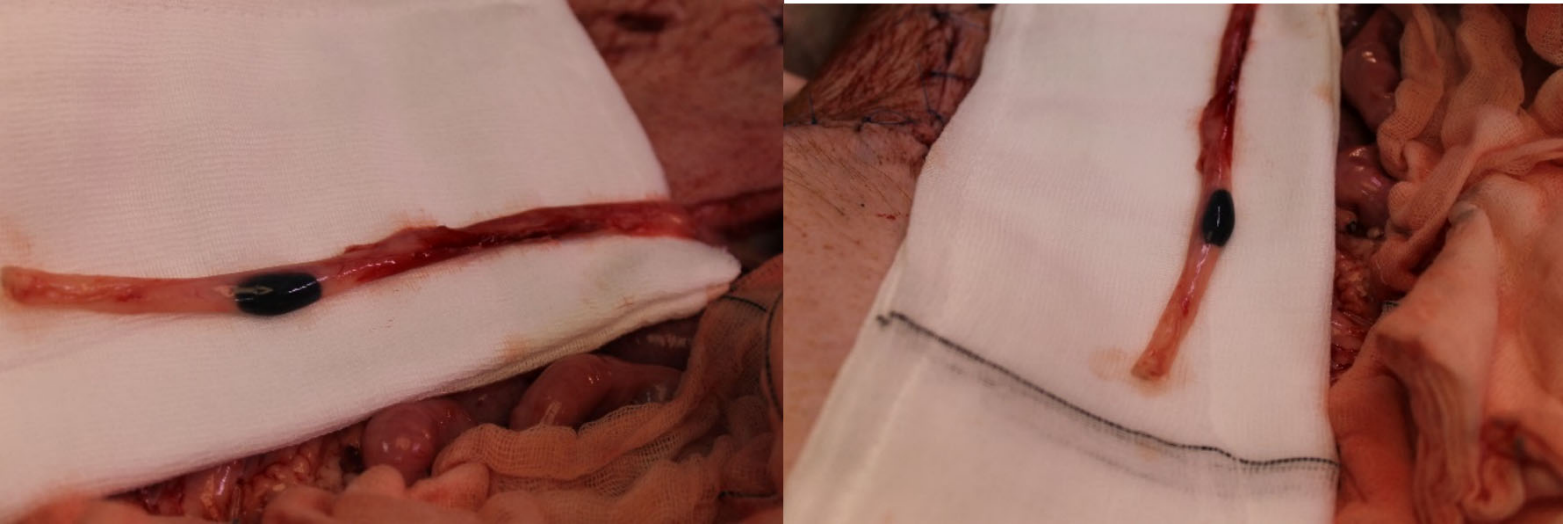
Illustrates the animal experiment on a living pig, showcasing the ureters with effective ink marking at dosages ranging from 0.1 to 0.2 ml.

We started with a dosage of 1 ml and gradually reduced the amount to determine the lowest effective concentration that would still accurately mark the targeted area. Our findings revealed that ink volumes ranging from 0.1 to 0.2 ml were ideal. These dosages were sufficient to mark a precise 1 cm section of the ureter wall, ensuring the marked area remained confined and clearly visible from the outside. This level of precision is critical as it aids in conducting exact resections, making the procedure safer and more efficient (Figure 1).

### 2.2 Surgical procedures

#### 2.2.1 Ureteroscopy with Black Eye™ Endoscopic Marker Ink injection

The patient is positioned in a dorsal lithotomy position, which provides optimal access for the procedure. Initially, a cystoscope is carefully inserted through the urethra into the bladder to locate the orifice of the affected ureter, a thin guidewire of a 0.038 French is threaded into it, serving as a safety guidewire. Following this, the cystoscope is removed, and a ureteroscope of 8.5 French, is inserted alongside the guidewire into the ureter. The ureteroscope is navigated towards the lower boundary of the tumour within the ureter.

Through the working channel of the ureteroscope, a injeTAK® Cystoscopy Needle, is introduced. This needle is used to inject a small amount of Black Eye™ Endoscopic Marker Ink, typically between 0.1 and 0.2 ml, at the lower edge of the tumour. If the ureteroscope can be manoeuvred past the tumour, a similar injection is made at the upper boundary of the tumour to ensure complete demarcation.

**FIGURE 2:**
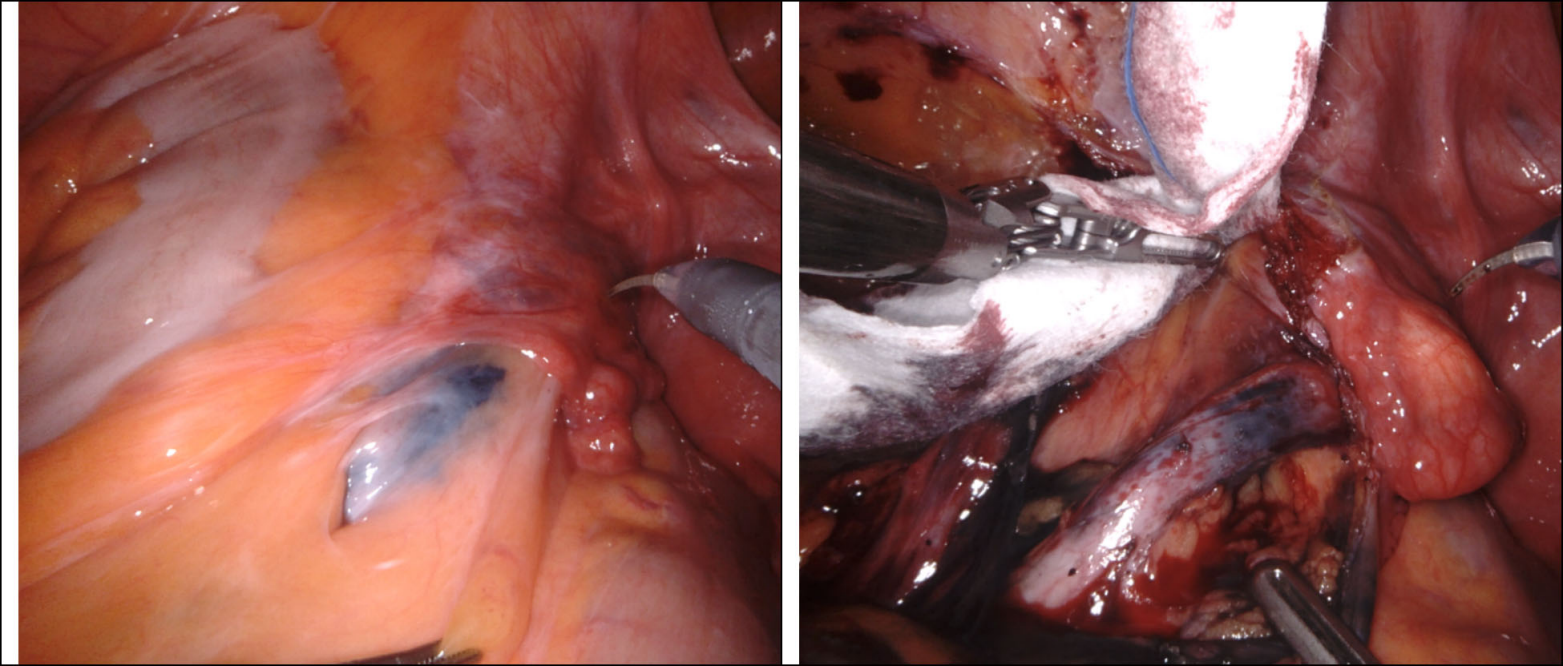
Black Eye™ Endoscopic Marker Ink marker on the left ureter shows the ink marker proximal to the tumour.

Upon completion of the ink injections, the procedure is concluded. The patient is then carefully repositioned to lie flat on their back, and preparations are made for the next phase of the surgical treatment (Figure 2).

#### 2.2.2 Robotic segmental resection

Initially, four robotic trocars, each 8 mm in size, are strategically placed in a line approximately 2 cm above umbilicus. Additionally, a 12 mm trocar is inserted to serve as an assistant port. This setup facilitates the insertion of robotic instruments and provides access for the surgical team.

The surgery begins with the identification of the ureter where it crosses the iliac artery, a key anatomical landmark. The peritoneum is carefully opened to expose the ureter. The ureter is then meticulously dissected free from surrounding tissues, allowing the surgeon to locate the previously applied Black Eye™ Endoscopic Marker Ink markers.

The tumour is securely isolated by placing hemo‐o‐lok clips, both 1 cm below and above the marked areas with Black Eye™ Endoscopic Marker Ink. This ensures that the entire section of the ureter containing the tumour is clearly identified and isolated.

The subsequent steps depend on the tumour's location along the ureter. For tumours located near the lower end of the ureter, a distal ureterectomy is performed, which involves removing the affected segment of the ureter. The remaining healthy portion of the ureter is then reimplanted directly to the bladder. In cases where the tumour is situated above the level of the iliac vessels, the end‐to‐end anastomosis was performed. This technique involves removing the tumour‐bearing segment and then meticulously suturing the two healthy ends of the ureter.

The specimen is carefully placed in an Endocatch bag.

Additionally, local and iliac lymph node dissection is performed as part of the procedure for all patients.

#### 2.2.3 Follow‐up regime

Patients who underwent SU are enrolled in a detailed follow‐up program to ensure they are recovering well and to check for any signs of recurrences or complications from the surgery. This program includes CT urography scheduled at 4, 8 and 12 months after surgery. Alongside these scans, patients also undergo a cystoureteronephroscopy. After the initial two years, the frequency of CT urography is reduced to once a year and continues for a total of five years. This careful monitoring is crucial for catching any issues early and ensuring the best possible outcome for the patient.

### 2.3 Statistical Analysis and Matching Process

In the study comparing outcomes between SU procedures in 30 patients and RNU in 130 patients, we used propensity score matching. This approach helps to ensure that the two groups being compared are as similar as possible, aside from the treatment they received. By matching patients from each group based on tumour size and location, we aimed to minimise any biases that could affect the study's outcomes.

Propensity score matching involves using statistical techniques, like logistic regression, to calculate a score for each patient. This score reflects the likelihood of a patient undergoing one type of surgery over the other, based on the characteristics selected for matching. Once these scores are calculated, patients from the SU group are matched with those in the RNU group who have similar scores, allowing for a more balanced and fair comparison between the two surgical methods. This method leads to a match of 40 patients from the control group.

To analyse the data from these matched groups, we used various statistical tests tailored to the nature of the data. Continuous variables that were normally distributed were analysed with Student's t‐tests. For continuous variables not following a normal distribution, the Mann–Whitney U test was employed, comparing the median values. Categorical variables were analysed using Fisher's exact test or the Chi‐square test, depending on the size of the data set and the distribution of values within categories.

All statistical analyses performed by the statistic program STATA 17 software.

## 3 RESULTS

After propensity score matching, the RUN arm comprised 40 patients, while the SU arm included 30 patients. The demographic and clinical profiles of the patients were found to be similar across the two groups studied. In this comparison, the RNU group consisted of relatively younger patients with an average age of 71.5 years, while those undergoing SU procedures had an average age of 77.6 years, *p* < 0.001. However, the gender distribution was fairly consistent between the two groups, with a majority being male (RNU: 30/40 males; SU: 22/30 males; *p* = 0.34). The median follow‐up time was 35 months (Table 1).

**TABLE 1:** Demographic and clinical characteristics of patients.

	RNU (*n* = 40)	SU (*n* = 30)	*p*
Age (years), mean (SD)	71.5 (6.7)	77.6 (3.2)	<0.001
**Gender, *n***			
‐Male	30	22	0.34
‐Female	10	8	
BMI	26.5 (5.2)	26.6 (5.3)	0.96
**Performance Score**			
0	19	12	0.7
1	15	14	
2	5	4	
3	1	0	
**Smoking, m**			
Active smoker	20	5	0.003
Previous smoker	12	21	
Never smoked	8	4	
Hydronephrosis, *n*	29	18	0.27
eGFR (IQR)	60 (49,78.5)	50 (44,70)	0.56
Previous bladder cancer, *n*	15	12	0.83
Stage of previous bladder cancer, *n*			
CIS	2	0	
Ta	12	12	
T1a	1	0	

Data presented as mean and standard deviation (SD) or median and Interquartile range (IQR) for continuous variables, and as *n* for categorical variables: There is no baseline missing data. RNU, Radical nephroureterectomy. SU, Segmental resection. BMI, Body mass index. Performance Score is based on the Eastern Cooperative Oncology Group (ECOG) scale. eGFR, estimated glomerular filtration rate (mL/min/1.73 m^2). CIS, Carcinoma in situ.

Regarding tumour‐specific features, there was no significant variance at the diagnostic biopsy stage (*p* = 0.29). In the RUN arm, nine patients had inconclusive biopsies, compared to three patients in the SU arm. Tumour size, evaluated by CT scans, presented a noticeable difference; RNU patients had larger tumours (median size 42.5 mm, IQR: 30–60.5) compared to those undergoing SU (median size 30 mm, IQR: 20–35) (*p* = 0.007). In the RUN arm, there were 14 patients with tumours located in the middle of the ureter, while in the SU arm, there were 9 patients with this tumour location (see Table 2).

**TABLE 2:** Surgical metrics and postoperative results for radical nephroureterectomy compared to segmental ureterectomy.

	RNU	SU	*p*
LN dissection, *n*	15	14	0.44
Positive LNs, *n*	1	0	0.96
Positive margin, *n*	4	1	0.31
**Stage post‐op, *n***			
Ta	20	24	0.09
T1	5	2	
T2	7	5	
T3	8	2	
**Grade post‐op, *n***			
Low grade	20	22	0.72
High grade	8	6	
**Tumour location, *n***			
Middle ureter	14	9	0.65
Lower ureter	26	21	
Op time,min (SD)	191 (46.5)	150 (47)	<0.001
Intraop bleeding, ml (IQR)	0 (0,25)	0 (0,50)	0.95
Post op stay, days (range)	1 (1–11)	1 (1–5)	0.11
**Post‐op complication, *n***			
Fascial dehiscence	1	0	0.76
Reoperation	2	0	0.50
Infection	4	0	0.13
AKI	2	0	0.50
**Clavien Dindo complication grade**			
Grade 1	1	0	0.30
Grade 2	3	4	
Grade 3a	1	0	
Grade 3b	3	0	
Grade 5 (Death)	2	0	
**Recurrence**			
Local	3	1	0.63
Bladder	17	10	0.43
Overall mortality	8	2	0.17
CSM	4	2	0.62

Data are presented as number (*n*), median (Interquartile Range, IQR), mean (Standard Deviation, SD). RNU, Radical Nephroureterectomy. SU, Segmental Resection. LN dissection, whether lymph nodes were dissected. Positive LNs, presence of cancer in lymph nodes. Stage post‐op, tumour stage after operation according to TNM classification. Op time, duration of the operation in minutes. Intraop bleeding, blood loss during surgery in milliliters. Post‐op stay, length of hospital stay after the operation. Local, local tumour recurrence; bladder, bladder tumour recurrence. CSM, cancer‐specific mortality.

The surgical and postoperative metrics unveiled a significantly reduced operation time for SU, with the mean duration for RNU being 191 minutes, SD 46.5, compared to 150 minutes for SU, SD 46 (*p* < 0.001). It's noteworthy that the average time for performing Black Eye™ Endoscopic Marker Ink marking was 20–25 minutes for patients undergoing SU. No complications were reported related to the injection of the Black Eye™ Endoscopic Marker Ink.

Four patients in the RNU had a positive surgical margin, while only one patient had a positive margin in the SU group (*p* = 0.31). Three patients had a local recurrences after RUN and only one patient had local recurrences in the SU arm (*p* = 0.63). The incidence of postoperative complications, such as infections and acute kidney injuries (AKI), was higher in the RNU group, although this did not reach statistical significance. The Clavien‐Dindo complication grades were similar between the two procedures (*p* = 0.30). Recurrences, both local and bladder, were more frequent in the RNU group, but the difference was not statistically significant (Table 2).

Further, when evaluating body mass index (BMI) and performance scores according to the Eastern Cooperative Oncology Group (ECOG) scale, no notable differences were observed, indicating that both groups had comparable physical status and functionality. The estimated glomerular filtration rates (eGFR), before surgeries, showed slightly lower values in the SU group, although this difference was not statistically significant (*p* = 0.56). Additionally, the incidence of previous bladder cancer histories appeared similar between the groups (RNU: 15/40 patients; SU: 12/30 patients; *p* = 0.83).

## 4 DISCUSSION

Our study's findings indicate that SU with Black Eye™ Endoscopic Marker Ink marking is an effective KSS that potentially provides oncological control comparable to RNU, particularly in patients with localised UTUC. The novel application of Black Eye™ Endoscopic Marker Ink to demarcate resection margins uniquely contributes to the field, enhancing surgical precision and potentially reducing positive surgical margins. This is a noteworthy advance not yet described in the existing literature. Although our study cohort was relatively small, positive surgical margins were lower in the SU group (1 out of 30 patients) than in the RNU group (4 out of 40 patients). This is particularly significant given that prior research has established frozen section analysis (FSA) as a method to diminish the likelihood of positive surgical margins during SU and RNU.^9^, ^10^, ^11^ This outcome is particularly encouraging when considering the utility of Black Eye™ Endoscopic Marker Ink marking as a simpler and cost‐effective alternative to FSA, which has been traditionally used to minimise the risk of positive surgical margins. For instance, Huang et al. reported a reduced incidence of positive margins utilising FSA during SU. Our findings suggest that Black Eye™ Endoscopic Marker Ink could serve the same purpose without the associated time and resource demands of the FSA.^12^

Furthermore, our data concur with meta‐analyses which indicate that for patients with low‐grade, localised tumours, KSS may offer equivalent oncological outcomes to more radical surgeries while preserving renal function.^13^ The strategic shift towards KSS for managing high‐grade disease, as evidenced in six patients from our SU group, aligns with current literature advocating for less invasive treatments that maintain favourable outcomes. This trend towards local treatment for high‐grade disease necessitates a thorough discussion on the balance between the aggressiveness of the disease and the benefits of organ preservation, especially in terms of disease‐specific and overall survival.^10^

SU presents a substantial advantage over RNU regarding operative time, with SU procedures averaging 41 minutes shorter (*p* < 0.001). This reduction in operative time can significantly benefit patients by reducing their exposure to anaesthesia and decreasing the time spent in the operating room, which can be associated with lower perioperative risks. Additionally, as surgeons gain more experience with this new procedure, the learning curve could lead to further reductions in surgical time, enhancing efficiency and possibly improving patient outcomes.

The observed size discrepancy between tumours in the SU and RNU groups in our study (*p* = 0.007) also highlights the role of tumour size in surgical decision‐making, corroborating the meta‐analysis conclusions regarding the importance of patient selection in optimising UTUC management strategies.^13^

The major limitation of our study is the selection bias; the selection criteria for SU versus RNU may introduce bias, as patients with larger tumours often offered RNU. This could limit the comparability between groups and affect the generalisability of the results. Additionally, a small sample size might limit the statistical power to detect differences, particularly in subgroup analyses. Furthermore, while a median follow‐up of 35 months provides initial insights into recurrence rates and patient outcomes post‐surgery, longer‐term follow‐up would be necessary to fully understand the implications of SU versus RNU, especially concerning survival rates and long‐term renal function.

In conclusion, SU with Black Eye™ Endoscopic Marker Ink marking is a viable KSS technique that offers a safe and effective alternative to RNU for patient with a single tumour, no longer than 30 mm and of low grade. This novel approach shows promise in reducing the risk of positive margins and ensuring effective cancer control, without a significant increase in complications. Despite the brief follow‐up period and a small group of participants, the results indicate that SU with Black Eye™ Endoscopic Marker Ink marking maintains oncological safety without compromise.

## AUTHOR CONTRIBUTIONS

Hayder Alhusseinawi contributed to the conception, data collection and analysis and interpretation. Nessn Azawi, Thomas Norus and Juan Luis designed and managed the qualitative analysis, project administration, supervision, review and editing. Naomi Nadler ans Helene Reif Andersen contributed to data collection.

## CONFLICT OF INTEREST STATEMENT

Juan Luis Vásquez declares consulting fees: Ambu, Photocure, Patents planned, issued or pending: an electrode assembly for improved electric field distribution and endoscopic use in the urinary bladder, Participation on a Data Safety Monitoring Board or Advisory Board: Janssen, MSD, Lina Medical, Photocure. All other authors declare no conflicts of interest.
